# Isolated mediastinal lymphangioma in a child: A rare case report

**DOI:** 10.4102/sajr.v29i1.3274

**Published:** 2025-12-20

**Authors:** Ankita Gupta, Maheswar Chaudhury, Manoranjan Khuntia, Peeta H. Prasad, Somadatta Das

**Affiliations:** 1 Department of Radiodiagnosis, Institute of Medical Sciences and SUM Hospital, Siksha ‘O’ Anusandhan, Deemed to be University, Bhubaneswar, India

**Keywords:** benign vascular tumour, mediastinal lymphangioma, cystic lymphangiomas, cystic lesion, recurrent pleural effusion

## Abstract

The mediastinum is an unusual location for cystic lymphangiomas. An 18-month-old male presented with acute fever, dry intermittent cough and respiratory distress. Chest radiography, ultrasonography, contrast-enhanced CT and MRI suggested a diagnosis of mediastinal cystic lymphangioma with internal haemorrhage. Surgical excision of the lesion and histopathological examination confirmed cystic lymphangioma.

**Contribution:**

This case highlights the multimodal radiological features of isolated mediastinal cystic lymphangioma for accurate diagnosis and improved management, to avoid unnecessary interventions and complications.

## Introduction

Solitary cystic lymphangioma is an uncommon congenital benign vascular tumour caused by a malformation of the lymphatic vessels. Lymphangiomas can affect any site in the body, most commonly the cervical (75%) and axillary regions (20%). Less than 1% of lymphangiomas are mediastinal.^1^ Most mediastinal lymphangiomas are located in the anterior mediastinum.

Lymphangiomas, although benign, can present with complications such as infection, cystic haemorrhage, superior vena cava syndrome, airway compromise, chylothorax and chylopericardium.^2^ Landing and Farber classified lymphangiomas into three categories:

- simple or capillary lymphangioma – dilated, capillary-sized lymphatic vessels connected to a normal lymphatic network
- cystic lymphangioma – multiple large cyst-like spaces lined by flat endothelial cells; the cystic spaces may be empty or filled with clear proteinaceous or chylous fluid containing lymphocytes, or occasionally red blood cells
- cavernous lymphangioma – dilated lymphatic sinuses in an actively growing lymphoid stroma, also connected to normal lymphatics.^3,4^

A compilation of clinical presentation, radiological imaging and histopathological investigation aids in diagnosing lymphangioma. A case of anterior mediastinal cystic lymphangioma with chief complaints of fever, intermittent cough and excessive crying in an 18-month-old male is presented.

## Ethical considerations

Written informed consent was obtained from the legal guardian of the patient.

## Patient presentation

An 18-month-old male was admitted to a tertiary care hospital for persistent fever and intermittent dry cough associated with excessive crying. He was delivered at term as the 1st order twin (the 2nd twin was stillborn), requiring admission to the neonatal intensive care unit for early-onset sepsis on day 2 of life, for which he received antibiotics and was discharged with no subsequent illness documented. On admission, physical examination revealed decreased air entry in the left mammary, infra-mammary and infra-axillary regions. The patient was febrile and irritable, with a per-abdominal examination suggesting hepatomegaly. An initial suspicion of meningitis was made, for which IV fluids and empiric antibiotics were commenced. Examination of the CSF revealed two white cells/*µ*L, excluding meningitis.

**FIGURE 1:**
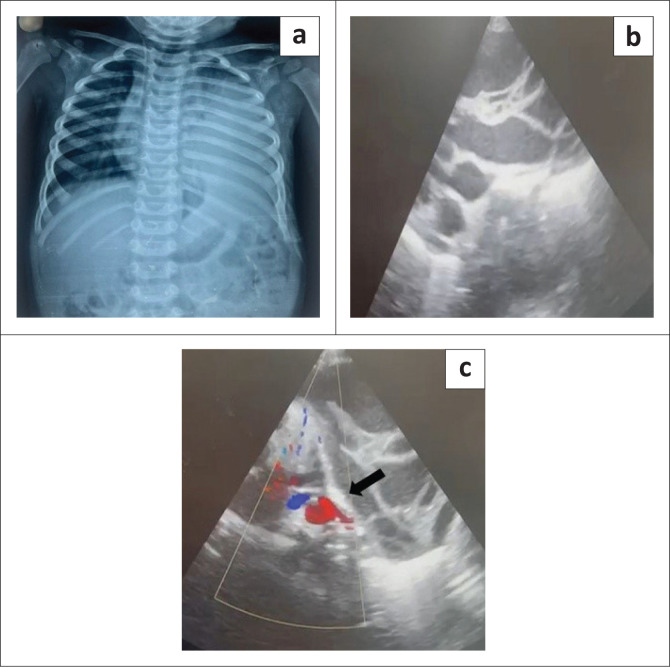
(a) Chest radiograph revealing a large homogeneous opacity in the left hemithorax silhouetting the left cardiac margin and ipsilateral hemidiaphragm, and a widened mediastinum. (b) Ultrasound demonstrating a well-defined, multiseptated cystic lesion in the left hemithorax. (c) Doppler ultrasound indicates the lesion abutting the pericardium (black arrow).

**FIGURE 2:**
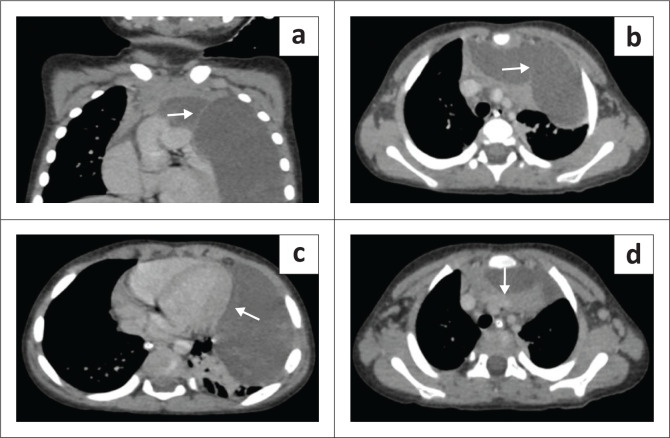
(a, b) Contrast-enhanced CT (CECT) revealing a large, non-enhancing, cystic lesion without a perceptible wall, occupying almost the entire left hemithorax, extending to the superior mediastinum and demonstrating non-enhancing septations (white arrows). (c) The lesion is abutting the pericardium (white arrow). (d) Axial CECT shows the lesion indenting the anterior wall of the thymus (white arrow).

**FIGURE 3:**
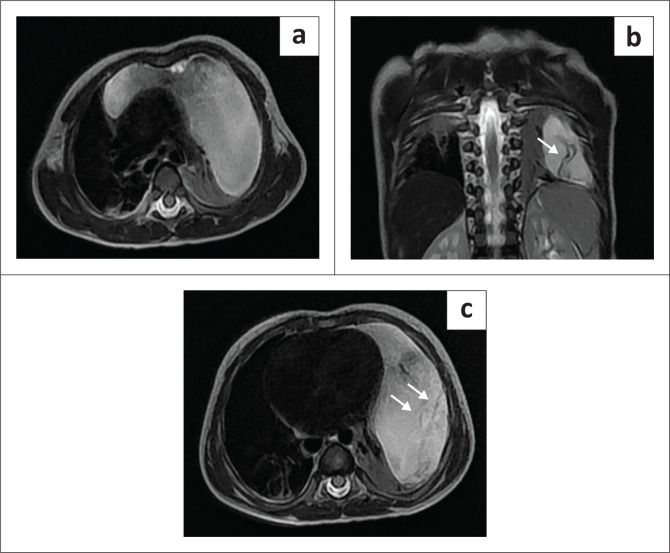
(a) Axial T2 MRI showing a large, hyperintense, cystic lesion in the anterior mediastinum and left hemithorax, compressing the left lung. (b, c) Coronal and axial T2-weighted MRI demonstrates septations within the lesion (white arrows).

**FIGURE 4:**
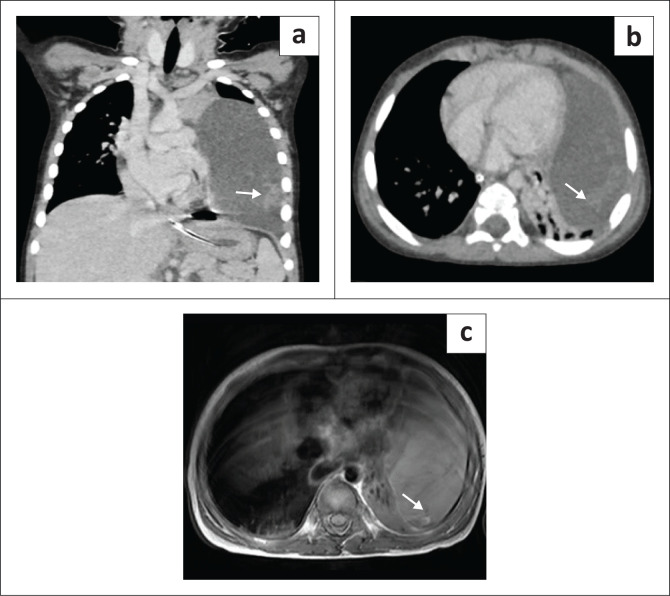
(a, b) Contrast-enhanced CT (CECT) coronal and axial images show hyperdense content within the lesion, suggestive of haemorrhage (white arrows). (c) Axial non-contrast T1-weighted MRI shows hyperintensity, indicating haemorrhage (white arrow).

Chest radiography revealed a homogeneously opacified left hemithorax, silhouetting the ipsilateral cardiac margin and hemidiaphragm, and a widened mediastinum (Figure 1a). Ultrasound with Doppler of the thorax showed a well-defined, multiseptated, cystic lesion in the left hemithorax measuring 8 × 4 cm, abutting the pericardium (Figure 1b and Figure 1c). Contrast-enhanced CT (CECT) of the thorax with angiography was advised to exclude congenital pulmonary airway malformation (CPAM). Imaging with CT revealed a large, non-enhancing, septated, cystic lesion measuring 8.7 × 8.5 × 6.4 cm,without a perceptible wall, in the anterior mediastinum and left hemithorax (Figure 2a and Figure 2b). The mass extended to the superior mediastinum but did not involve the neck, abutting the right mediastinal pleura and pericardium and indenting the anterior wall of the thymus, causing secondary collapse of the left lung basal segments and deviation of the trachea to the right (Figure 2c and Figure 2d). The MRI showed a large T2 hyperintense cystic lesion with thin septations in the anterior mediastinum and left hemithorax compressing the left lung, consistent with the CECT findings (Figure 3). Dependent, non-enhancing, hyperdense content within the lesion on the CT scan corresponded to T1 hyperintensity on the MRI, likely indicating haemorrhage (Figure 4).

**FIGURE 5:**
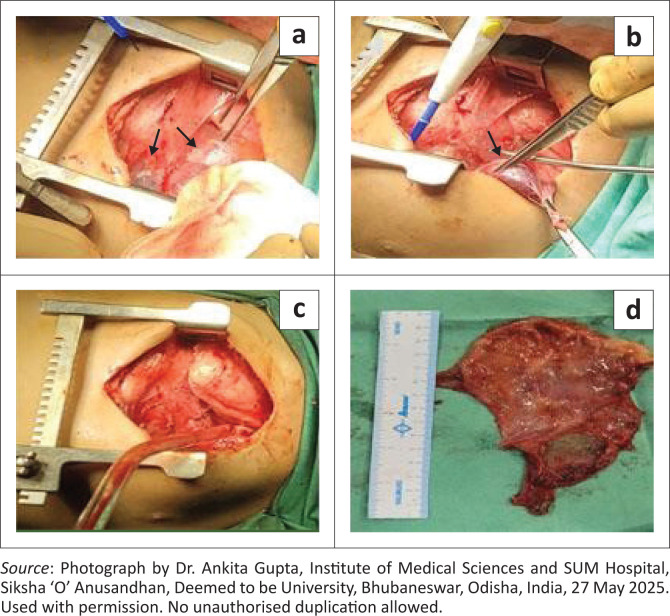
(a, b) Anterior mediastinal cystic mass (black arrows), (c) post-excision, and (d) excised cystic lymphangioma.

**FIGURE 6:**
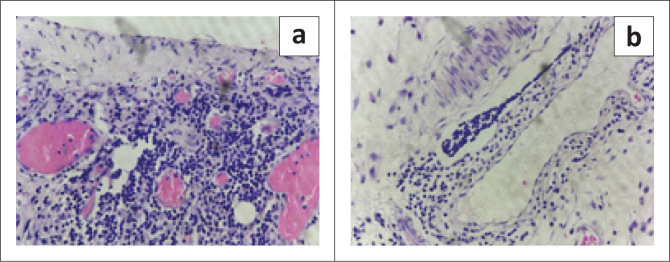
(a) Cystic space revealing a dense lymphoid aggregate and proliferated capillaries. (b) Dilated lymphatic channels containing mature lymphocytes.

Following a suggested diagnosis of mediastinal cystic lymphangioma complicated by internal haemorrhage, median sternotomy with excision of the anterior mediastinal cystic mass (Figure 5) was performed under general anaesthesia. Histopathological examination revealed fibro-adipose tissue, cystic spaces lined by a corrugated, single layer of cuboidal epithelium and adjacent fibrovascular connective tissue showing dense lymphoplasmacytic infiltrates and scattered, dilated mature lymphocytes, suggesting a benign vascular malformation compatible with lymphangioma (Figure 6). The patient developed a Candida infection at the central line site on day 10 post-surgery, for which antifungals and antibiotics were administered. The patient was discharged on day 12 post-surgery.

## Discussion

Cystic lymphangiomas most commonly develop superficially on the body surface and are often detected before the age of 2 years.^1,5^ Only 2% – 3% of the cervical lymphangiomas may be associated with an intrathoracic extension. An isolated mediastinal lymphangioma without a cervical component is rare, accounting for less than 1%.^5^ The child in the presented case had an isolated mediastinal cystic lymphangioma without any cervical component.

Cystic lymphangiomas are usually asymptomatic until they reach dimensions large enough to cause compression of the adjacent structures, leading to respiratory distress and causing internal haemorrhage at a later stage of progression.^6,7^ The index patient presented with respiratory distress, which required supportive treatment pre-operatively.

The differential diagnosis of an anterior mediastinal lymphangioma includes bronchogenic cyst (well defined, non-enhancing, homogeneous cystic lesion with thin smooth walls in the carinal, paratracheal, oesophageal or retrocardiac areas); pericardial cyst; duplication cyst (features same as bronchogenic cyst with their location being the differentiating feature); necrotic neoplasm (thick- or irregular-walled mass on CT); thymic cyst (imperceptible, thin-walled unilocular cyst if congenital and multiloculated cyst associated with thymic neoplasias if acquired); haematoma, seroma or abscess (low-attenuation mass with an enhancing rim); as well as mature teratoma (cystic mediastinal mass with a combination of multiple tissue elements – fat and cystic components or fat-fluid level within the mass on CT and a heterogenous mediastinal mass with a variable mixture of fat, fluid, soft tissue and calcification on MRI).^8^ Shape, cyst wall thickness, intracystic septations, presence of a solid component, fat, or calcification, and infiltration of surrounding structures are the differentiating criteria between benign congenital cysts and other cyst-like lesions.^9^

Lymphangiomas can also mimic recurrent pleural effusion, which may lead to unnecessary interventional procedures. This further highlights the importance of multimodal imaging investigations for the assessment and diagnosis of lymphangiomas. Although unusual, lymphangiomas should be considered as a differential in cases of recurrent thoracic fluid accumulation.^10,11,12^ Ultrasound, being a non-invasive, cost-effective and non-radiation imaging modality, is considered the first level investigation for a mass suspicious of cystic lymphangioma. It is then integrated with higher modalities such as CT and MRI to obtain additional information such as structural features, internal and peripheral contrast enhancement, as well as loco-regional lesion spread. Radiological imaging plays an important role in excluding malignancy and providing the exact anatomic location of the mass before surgery.^13^

Surgical excision of the mass is the treatment of choice and a confirmatory diagnosis is made through histopathological examination.^7^ In the case presented, the patient underwent open sternotomy with excision of the space-occupying lesion under general anaesthesia. Post-operative complications include infection, chylothorax, fistula formation, injury to the phrenic nerve, vagus nerve, lungs, or major vessels.^7^ In the index patient, the whole tumour was resected successfully. The chances of recurrence are lower following a complete resection, which is otherwise relatively common (35% vs. 6% recurrence in the case of complete resection).^14^ Follow-up is therefore indicated for any event of recurrence. If complete surgical resection is deemed inconvenient, as in cases of large tumour size, mediastinal or neurovascular bundle infiltration, or multiple loculations, chemotherapy, radiotherapy, and sclerotherapy combined with partial resection can be considered as alternative treatments with varied results.^12,15^

## Conclusion

This report describes a case of an isolated mediastinal cystic lymphangioma without any cervical component, contributing to existing literature based on its rarity and unusual location.
